# A Comprehensive Computer-Assisted Diagnosis System for Early Assessment of Renal Cancer Tumors

**DOI:** 10.3390/s21144928

**Published:** 2021-07-20

**Authors:** Mohamed Shehata, Ahmed Alksas, Rasha T. Abouelkheir, Ahmed Elmahdy, Ahmed Shaffie, Ahmed Soliman, Mohammed Ghazal, Hadil Abu Khalifeh, Reem Salim, Ahmed Abdel Khalek Abdel Razek, Norah Saleh Alghamdi, Ayman El-Baz

**Affiliations:** 1 BioImaging Lab, Bioengineering Department, University of Louisville, Louisville, KY 40292, USA; mnsheh01@louisville.edu (M.S.); ammost01@louisville.edu (A.A.); amshaf02@louisville.edu (A.S.); ahmed.soliman@louisville.edu (A.S.); 2 Department of Radiology, Urology and Nephrology Center, University of Mansoura, Mansoura 35516, Egypt; rashataha2020@gmail.com (R.T.A.); ahmed.elmahdy89@yahoo.com (A.E.); 3 College of Engineering, Abu Dhabi University, Abu Dhabi 59911, United Arab Emirates; mohammed.ghazal@adu.ac.ae (M.G.); hadil.abukhalifeh@adu.ac.ae (H.A.K.); reem.salim@adu.ac.ae (R.S.); 4 Department of Radiology, Faculty of Medicine, Mansoura University, Mansoura 35516, Egypt; arazek@mans.edu.eg; 5 College of Computer and Information Science, Princess Nourah Bint Abdulrahman University, Riyadh 11564, Saudi Arabia; nosalghamdi@pnu.edu.sa

**Keywords:** renal cell carcinoma, CE-CT, morphology, texture, functionality, RC-CAD

## Abstract

Renal cell carcinoma (RCC) is the most common and a highly aggressive type of malignant renal tumor. In this manuscript, we aim to identify and integrate the optimal discriminating morphological, textural, and functional features that best describe the malignancy status of a given renal tumor. The integrated discriminating features may lead to the development of a novel comprehensive renal cancer computer-assisted diagnosis (RC-CAD) system with the ability to discriminate between benign and malignant renal tumors and specify the malignancy subtypes for optimal medical management. Informed consent was obtained from a total of 140 biopsy-proven patients to participate in the study (male = 72 and female = 68, age range = 15 to 87 years). There were 70 patients who had RCC (40 clear cell RCC (ccRCC), 30 nonclear cell RCC (nccRCC)), while the other 70 had benign angiomyolipoma tumors. Contrast-enhanced computed tomography (CE-CT) images were acquired, and renal tumors were segmented for all patients to allow the extraction of discriminating imaging features. The RC-CAD system incorporates the following major steps: (i) applying a new parametric spherical harmonic technique to estimate the morphological features, (ii) modeling a novel angular invariant gray-level co-occurrence matrix to estimate the textural features, and (iii) constructing wash-in/wash-out slopes to estimate the functional features by quantifying enhancement variations across different CE-CT phases. These features were subsequently combined and processed using a two-stage multilayer perceptron artificial neural network (MLP-ANN) classifier to classify the renal tumor as benign or malignant and identify the malignancy subtype as well. Using the combined features and a leave-one-subject-out cross-validation approach, the developed RC-CAD system achieved a sensitivity of 95.3%±2.0%, a specificity of 99.9%±0.4%, and Dice similarity coefficient of 0.98±0.01 in differentiating malignant from benign tumors, as well as an overall accuracy of 89.6%±5.0% in discriminating ccRCC from nccRCC. The diagnostic abilities of the developed RC-CAD system were further validated using a randomly stratified 10-fold cross-validation approach. The obtained results using the proposed MLP-ANN classification model outperformed other machine learning classifiers (e.g., support vector machine, random forests, relational functional gradient boosting, etc.). Hence, integrating morphological, textural, and functional features enhances the diagnostic performance, making the proposal a reliable noninvasive diagnostic tool for renal tumors.

## 1. Introduction

Renal cancer is one of the most common malignancies, being the sixth most prevalent type of cancer among men and the eighth most prevalent among women. For the past several decades, an increasing number of new patients have been diagnosed with renal cancer. The year 2020 saw approximately 74,000 diagnoses of renal cancer in the United States [1,2], and 15,000 patients are expected to have died from renal cancer in that same time period [1,2]. Roughly two thirds of the time, renal cancer is diagnosed before it has metastasized, in which case the 5 y survival rate is 93%. Once it has spread to the lymph nodes or the surrounding abdominal structures (i.e., other organs or tissues), the 5 y survival rate falls to 70%. In the worst case of metastasis to distant parts of the body, the 5 y survival rate is a mere 12% [1,2]. In addition, the National Cancer Institute had an approximated cost estimate of $5.1 billion for renal cancer care in the United States by the end of 2020 [3].

Renal cancer is a heterogeneous disease in which the renal cells become malignant (cancerous) and form tumors called renal masses. These renal masses, if not detected early and treated promptly, will lead to mortality. The most common, and also the most aggressive, renal cancer is renal cell carcinoma (RCC), accounting for 70% of all cases [4,5]. In turn, 70% of RCC are clear cell renal cell carcinoma (ccRCC), and of the remaining nonclear cell subtypes (nccRCC), the most prevalent are papillary (paRCC) and chromophobe (chrRCC) renal cell carcinomas, accounting for 15% and 5% of all RCC, respectively [6]. The World Health Organization (WHO) taxonomy of RCC [6] has clinical significance because the various subtypes can have very different prognoses [6,7,8]. Differential diagnosis of RCC must look out for the benign tumors angiomyolipoma (AML) and oncocytoma (ONC), which are easily confused with RCC using conventional diagnostic techniques [9,10,11,12,13]. AMLs with low fat content are particularly prone to misdiagnosis [14]. Diagnostic error leads to unnecessary surgical intervention for benign lesions, to the point where 15–20% of surgically resected “RCC” may actually be AML [15]. Therefore, accurate characterization of such renal masses at an early stage is crucial to the identification of appropriate intervention plans and/or treatment courses.

### 1.1. Current Diagnostic Techniques and Their Limitations

Evidence of renal cancer can be found in complete blood count (CBC) to check for the number of red blood cells; urine tests to look for blood, bacteria, or cancerous cells in urine; and blood chemistry tests to quantify renal function by checking the levels of certain chemicals in the blood. These signs are suggestive at best, and inadequate for diagnosis or typing of renal cancer. Only biopsy, performed using interventional radiology, can provide a definite diagnosis of renal cancer, and thus remains the gold standard [1,2]. However, it can only be used as the last resort due to its high invasiveness, cost, and turnaround and recovery times (approximately a week). Therefore, the investigation of noninvasive imaging modalities (e.g., computed tomography (CT), magnetic resonance imaging (MRI), and ultrasounds) to provide an early, reliable, accurate, cost-effective, and rapid diagnosis of renal tumors is underway [16,17,18,19].

### 1.2. Related Work

One of the most important diagnostic imaging modalities for the accurate diagnosis of renal tumors is contrast-enhanced CT (CE-CT) [20,21]. Besides specifying the location, shape, and size of a tumor, CE-CT can also distinguish RCC from benign lesions with 77–84% accuracy based on their different uptake of the contrast agent [18,22,23]. For this purpose, texture analysis (TA) is performed on the CE-CT images to extract quantitative features [24,25]. As a radiomic technique, TA has seen an array of applications in typing, staging, and grading tumors and even in predicting treatment response and survival rates [24]. A recent study by Deng et al. [26] utilized TA techniques along with CE-CT to discriminate malignant from benign renal tumors. Their study included 501 renal tumors of which 354 were RCCs and 147 were benign lesions. From the portal-venous phase, they manually placed a region of interest (ROI) in the largest CE-CT cross-section of the tumor volume. Then, they extracted four textural features, namely entropy, kurtosis, mean positive pixel density, and skewness. Utilizing logistic regression, they found that higher values of entropy were significantly associated with a greater likelihood of malignancy (*p* = 0.022). As a diagnostic indicator of RCC, the entropy feature had high specificity (85.5%), but quite low sensitivity (31.3%) [26].

Another study was conducted by Kunapuli et al. [27] to explore the potential of CE-CT along with TA to identify malignant renal tumors. Their dataset included images of 100 malignant (70 ccRCC, 20 paRCC, and 10 chrRCC) and 50 benign (20 AML and 30 ONC) tumors. After segmenting renal tumors manually using image-rendering software, 2D and 3D TAs were performed on tumor with the largest diameter and the entire tumor volume, respectively. Fifty-one 2D and 3D textural features were extracted from each of four different CT phases, yielding a total of two-hundred and four features per subject. These comprised 8 histogram features (i.e., first-order textural features), 40 s-order textural features (20 grey-level co-occurrence matrix (GLCM) and 20 grey-level difference matrix (GLDM)), and 3 spectral features derived from the 2D Fourier transform. Recursive feature elimination [28] was used to reduce the number of features to 10 per phase, or a total of 40. Their classification algorithm incorporating these features, using relational functional gradient boosting, had a reported 82% accuracy and an 0.83 area under the curve. The classifier was developed to discriminate between malignant and benign tumors only, and the authors did not investigate the subtype classification of malignant RCC [27].

Kocak et al. [29] conducted a study to classify ccRCC renal tumors from nccRCC ones using CE-CT along with TA. A total of 68 RCCs were included for internal validation (N = 48 ccRCC and N = 20 nccRCC). For external validation purposes, they included an additional 26 RCC from a public dataset (N = 13 and N = 13 nccRCC). Their study utilized MaZda image-rendering software [30] to manually segment renal tumors on the largest/middle cross-section. This was followed by an extraction of 275 textural-related features from each subject in both the enhanced CT phase and the unenhanced phase. In addition, a wrapper-based nested cross-validation approach was employed to select the reproducible features in both phases and to optimize their classification model. Artificial neural networks (ANNs) were used, and a classification accuracy of 86.7%, a sensitivity of 80%, and a specificity of 89.6% on internal data and an accuracy of 84.6%, a sensitivity of 69.2%, and a specificity of 100% on external data were reported in differentiating ccRCC from nccRCC. Although their study reported a good overall classification performance between ccRCC and nccRCC, they were limited by their low sensitivity. In addition, they reported a very poor diagnostic performance to differentiate chrRCC from paRCC and from ccRCC. They suggested that CE-CT is more powerful at providing useful textural features than the unenhanced CT.

A bigger study was performed by Sun et al. [31] to compare between the diagnostic performance of machine learning approaches and four expert radiologists in differentiating malignant from benign renal tumors, as well as ccRCC from nccRCC malignant tumors using CE-CT. Their study included 254 malignant tumors (ccRCC = 190, nccRCC = 64 (chrRCC = 38, paRCC = 26)), 26 AML benign tumors, and 10 ONCs. After performing manual delineation of the tumor lesions, they used open-source software packages to extract and analyze textural features and used another open-source software to complete their analysis. Then, they utilized a support vector machine (SVM) classifier with a radial basis function along with a 10-fold cross-validation approach to obtain the final diagnosis. They reported sensitivities of 90%, 86.3%, and 73.4% using SVM compared to 73.7–96.8%, 73.7–96.8%, and 28.1–60.9% obtained by the 4 expert radiologists in differentiating ccRCC from nccRCC, ccRCC from AML and ONC, and nccRCC from AML and ONC, respectively. Hence, they concluded that machine learning approaches along with textural features have potential power, as well as low-variance performance in diagnosing renal tumors.

Lee et al. [32] used TA and CE-CT in their study to differentiate between ccRCC malignant and AML benign renal tumors. Their study included 80 renal tumors (ccRCC = 41 and AML = 39). They combined several hand-crafted textural features extracted from a 2D manually annotated central image of the entire mass with automated deep features extracted by different ImageNet pretrained convolutional neural network (CNN) classification models, namely AlexNet [33], VGGNet [34], GoogleNet [35], and ResNet [36]. Then, they used the combined features to train and test a random forest (RF) classifier. Using a leave-one-out cross-validation approach, their combined model achieved a diagnostic accuracy of 76.6% ± 1.4%, outperforming the individual diagnostic results using either the hand-crafted features alone or the deep features alone.

Oberai et al. [37] investigated the potential power of CNN along with multiphasic CE-CT images to differentiate benign from malignant renal masses. Their study included 143 patients (malignant = 97 and benign = 46). After performing manual segmentation of the whole tumor volume, they selected the largest axial segmented tumor image from each CE-CT phase to input in the CNN for training and validation. Using an 8-fold cross-validation approach, they reported an accuracy of 78%, a sensitivity of 70%, and a specificity of 81%. However, their dataset had an approximately 2:1 class imbalance, which might contribute to the reduced diagnostic performance. Although their study included different types of malignant tumors, they did not investigate the subtyping of malignant class.

Zhou et al. [38] conducted a study to distinguish between malignant and benign renal tumors using CE-CT along with an ImageNet-pretrained InceptionV3 model. This model was then cross-trained using transfer learning on their own dataset of 192 renal tumors (malignant: ccRCC = 117 and nccRCC = 17, benign: renal cyst = 50 and AML = 8). Several image-level models were considered, using whole CT slices, ROIs, and rectangular subregions of the CT-CT data. Then, during the transfer learning, different number of layers were frozen, resulting in two-patient level models based on the optimal image-level models. Using a five-fold cross-validation approach, they reported a 69% accuracy using the slice dataset, a 97% accuracy using the ROI dataset, and a 93% accuracy using the RBR dataset. In spite of achieving a high accuracy in differentiating malignant from benign renal tumors, 50 out of 58 benign cases were renal cysts, which are much easier to distinguish from RCC compared to AML. In addition, they did not investigate discriminating ccRCC from nccRCC renal tumors.

Shehata et al. [39] published a recent study to differentiate malignant RCC from benign AML renal tumors, as well as to identify the malignant RCC subtype using CE-CT. Their data included 105 biopsy-confirmed cases (ccRCC = 40, nccRCC = 30, and AML = 35). After performing manual segmentation to delineate the renal tumor, they extracted 22 first- and second-order textural features, as well as two functional features represented by wash-in and wash-out slopes. These features were subdivided into four groups. To differentiate RCC from AML, they obtained four preliminary diagnoses using separate RF classifiers on each feature group, then used weighted-majority voting to produce the final diagnosis. They reported a 96% accuracy, a 100% sensitivity, and an 89% specificity. Subsequently, for cases diagnosed as RCC, they utilized SVM classifiers along with the weighted-majority voting technique to specify the subtype of malignancy as ccRCC or nccRCC, for which the reported accuracy was 71.4%. In spite of correctly identifying 70 of 70 RCC cases, their system was not specific enough. This could be a consequence of the imbalance between the RCC and AML group sizes. In addition, their technique did not achieve a sufficient diagnostic performance in malignancy subtyping.

**Figure 1:**
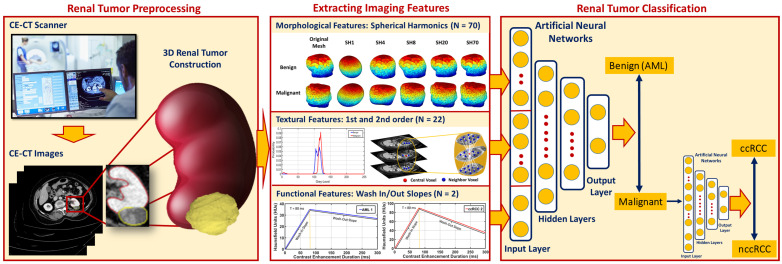
The proposed renal cancer computer-assisted diagnosis (RC-CAD) system.

Most of the studies referenced above were pure applications of TA to CE-CT imaging. That is to say, they did not integrate other features (e.g., morphological and functional) with two- or three-dimensional textural features to diagnose RCC. Only a few studies addressed typing of RCC, i.e., discrimination between ccRCC and nccRCC, which is vital information for deciding the course of treatment from the beginning. To overcome these limitations, we developed RC-CAD, a two-stage system for comprehensive computer-assisted diagnosis of renal cancer based on CE-CT imaging. RC-CAD (Figure 1) incorporates 3D morphological features, first- and second-order 3D textural features, and time-dependent metrics of renal function (wash-in/-out slopes) to provide a high diagnostic accuracy of cancerous renal tumors. The developed RC-CAD system has the ability to (i) discriminate malignant (RCC) from benign (AML) renal tumors and (ii) specify the subtype of malignant tumors as ccRCC vs. nccRCC. To the best of our knowledge, the developed CE-CT-based RC-CAD system is unique with the ability to integrate 3D morphological features with 3D textural features and functional features for early discrimination of RCC malignant tumors from AML benign tumors and determine the subtype of malignancy as ccRcc or nccRCC.

It is worth noting that this paper extends our recent work [39] with the following substantial modifications: (i) increasing the sample size from 105 (70 RCC vs. 35 AML) to 140 renal tumors (70 RCC vs. 70 AML) to ensure data balancing and to avoid any possible classification bias towards the majority class, (ii) applying a new parametric spherical harmonic technique to estimate the morphological features from the segmented renal tumors to capture the surface complexity/irregularity between different types of renal tumors, (iii) integrating/concatenating the estimated morphological features with the first- and second-order textural features and functional features, and (iv) modeling a two-stage classification using a multilayer perceptron artificial neural network (MLP-ANN) whose inputs comprise all the aforementioned discriminant features. The first stage decides if the renal tumor is malignant (RCC) or benign (AML). In the former case, the second stage identifies the malignancy subtype as ccRCC or nccRCC.

## 2. Materials

Patients who had undergone renal biopsy for suspected cancer (N=140) ranged from 15 to 87 years of age (mean = 50.5 years and standard deviation = 13.4 years). There were 72 patients who were males, while the remaining 68 were female. Informed consent was obtained from the patients themselves or their parents/legal guardians (age < 18 years) to participate in this study. Biopsy reports confirmed that 70 patients had RCC (40 ccRCC and 30 nccRCC, of which 17 were paRCC and 13 were chrRCC), while the other 70 had benign AML tumors. Study participants had undergone a multiphase CT examination prior to biopsy. Imaging was performed with a Brilliance CT 64 multislice scanner (Philips Medical Systems, Best, The Netherlands). A mechanical injector was used to administer contrast agent into an antecubital vein with a dose of 120 mL at a rate of 4.0 mL/s. The abdomen scanning included three main phases: a precontrast phase, a portal-venous phase, and a delayed-contrast phase acquired at *t* = 0, *t* = 80, and *t* = 300 s, respectively. All images were acquired using the following parameters: slice thickness = 2.5 mm; pitch = 0.984; rotation time = 0.75 s.

## 3. Methods

The proposed RC-CAD system pipeline (see Figure 1) performs the following steps to obtain the final diagnosis: (i) constructs 3D models of renal tumors from manually segmented 2D ROIs, (ii) applies a new parametric spherical harmonic technique to estimate the morphological features of the tumor boundary, (iii) constructs a rotation-invariant gray-level co-occurrence matrix (GLCM) to extract the textural features of the tumor volume, (iv) estimates the wash-in/wash-out slopes inside the 3D region, and (v) performs two-stage classification using an MLP-ANN whose inputs comprise all aforementioned discriminant features. The first stage decides if the renal tumor is malignant (RCC) or benign (AML). These steps are presented in detail next.

### 3.1. Renal Tumor Preprocessing

**Figure 2:**
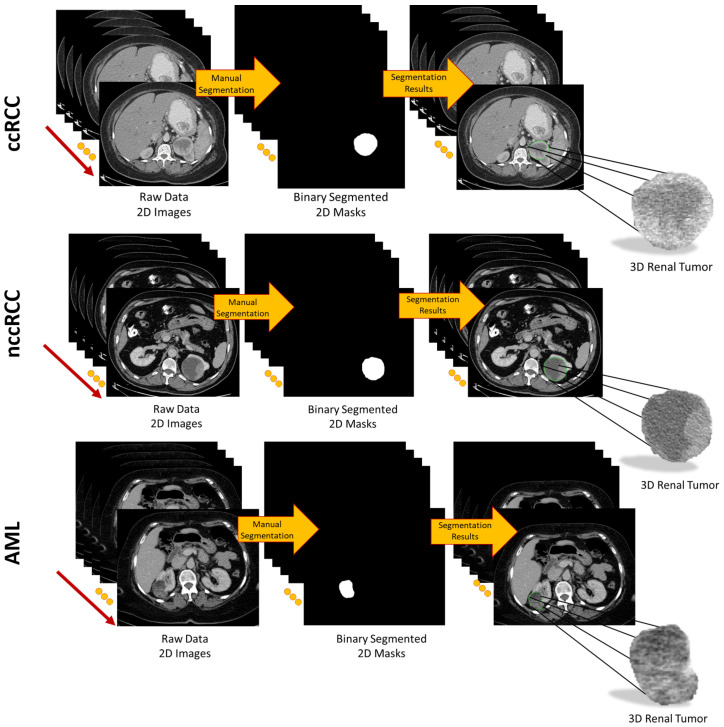
Visualization of the segmentation process to obtain 3D renal tumors.

To provide a more accurate extraction of morphological, textural, and functional discriminating imaging features, for each subject, each CT slice intersecting the renal tumor was accurately and manually segmented by expert radiologists to define the 2D ROI. Then, all 2D ROIs were stacked together to construct the 3D renal tumor object (3D ROI), as shown in Figure 2.

### 3.2. Extracting Imaging Features

For accurate identification of malignant renal tumors and the associated subtype, all 3D segmented volumes were characterized by their morphological, textural, and functional features, as described below.

**Figure 3:**
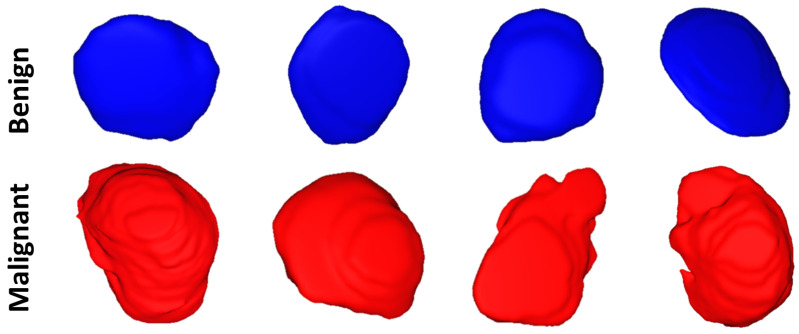
Visualizing 3D surface complexity differences between different renal tumors (benign are shown in blue, while malignant are shown in red).

**Morphological features:** To enhance both the sensitivity and specificity of early renal cancer diagnosis, morphological features of the tumor are incorporated into the algorithm. These features were designed to quantify the complex shape of the tumor boundary. This was motivated by the hypothesis that rapidly growing, malignant tumors develop more irregular/complex shapes relative to more slowly growing, benign tumors. Therefore, the utilization of such shape descriptors would enhance the performance of the automatic diagnosis. Examples of this phenomenon are illustrated in Figure 3.

Naturally, in order to measure the irregularity of the boundary, we must first construct an accurate shape model of the tumor. In this paper, we incorporated a state-of-the-art spectral decomposition in terms of spherical harmonics (SHs) [40] to construct this shape model. An arbitrary point in the interior of the tumor, or more specifically, the interior of its convex kernel, was selected as the origin (0,0,0). In this coordinate system, the tumor’s surface may be considered a function of the polar and azimuthal angle, f(θ,φ), which can be expressed as a linear combination of basis functions $Y_{\tau \beta }$ defined on the unit sphere. Starting with a discrete approximation of the surface, i.e., a triangular mesh, the proposed algorithm uses an attraction–repulsion technique [41] to map this mesh to the unit sphere. The mapping fixes the image of each mesh vertex at the unit distance from the origin, while preserving the mesh topology and maintaining the distance between adjacent vertices as much as possible.

Each iteration α of the attraction-repulsion works as follows. Let $C_{\alpha ,i}$ be the coordinates of the node on the unit sphere corresponding to mesh vertex *i* at the beginning of iteration α. Denote the vector from node *i* to node *j* by $d_{\alpha ,ji}=C_{\alpha ,j}-C_{\alpha ,i}$; then, the Euclidean distance between nodes *i* and *j* is $d_{\alpha ,ji}=∥d_{\alpha ,ji}∥$. Finally, let $J_{i}$ denote the index set of neighbors of vertex *i* in the triangulated mesh. Then, the attraction step updates the position of each node to keep it centered with respect to its neighbors:

(1) $$C_{\alpha +1,i}^{\prime }=C_{\alpha ,i}+C_{A,1}\sum_{j\in J_{i}}\left(d_{\alpha ,ji}d_{\alpha ,ji}^{2}+C_{A,2}\frac{d_{\alpha ,ji}}{d_{\alpha ,ji}}\right),$$

The quantities $C_{A,1}$ and $C_{A,2}$ are implementation-defined parameters that determine the strength of the attractive force. The next step, repulsion, inflates the spherical mesh to prevent it from degenerating (the attraction step by itself would allow nodes to become arbitrarily close to one another).

(2) $$C_{\alpha +1,i}^{\prime \prime }=C_{\alpha +1,i}^{\prime }+\frac{C_{R}}{2I}\sum_{j=1;j\neq i}^{I}\frac{d_{\alpha ,ji}}{d_{\alpha ,ji}^{2}},$$

Just as the attraction step, the repulsion step uses an implementation-defined parameter $C_{R}$ to set the strength of the repulsive force. Subsequently, the nodes are projected back onto the sphere by giving them the unit norm, and these are their coordinates at the beginning of the next iteration, $C_{\alpha +1,i}=C_{\alpha +1,i}^{\prime \prime }/∥C_{\alpha +1,i}^{\prime \prime }∥$.

At the terminal iteration $\alpha_{f}$ of the attraction–repulsion algorithm, the surface of the renal tumor is in a one-to-one correspondence with the unit sphere. Each node $C_{i}=\left(x_{i},y_{i},z_{i}\right)$ of the original mesh is mapped to a corresponding point $C_{\alpha_{f},i}=\left(\sin\theta_{i}\cos\phi_{i},\sin\theta_{i}\sin\phi_{i},\cos\theta_{i}\right)$ with polar angle $\theta_{i}\in \left[0,\pi \right]$ and azimuthal angle $\phi_{i}\in \left[0,2\pi \right)$. Considering these points as samples of a continuous function f(θ,φ) defining the boundary, the tumor shape may be estimated by fitting an SH series to the sample nodes, since the SHs form an orthogonal basis for functions on a sphere. The SH $Y_{\tau \beta }$ of degree τ and order β is defined as:

(3) $$Y_{\tau \beta }=\left\{\begin{array}{cc} c_{\tau \beta }G_{\tau }^{\left|\beta \right|}\cos\theta \sin\left(\left|\beta \right|\varphi \right) & -\tau \leq \beta \leq -1\\ \frac{c_{\tau \beta }}{\sqrt{2}}G_{\tau }^{\left|\beta \right|}\cos\theta & \beta =0\\ c_{\tau \beta }G_{\tau }^{\left|\beta \right|}\cos\theta \cos\left(\left|\beta \right|\varphi \right) & 1\leq \beta \leq \tau \end{array}\right.$$

where $c_{\tau \beta }$ is the SH factor and $G_{\tau }^{\left|\beta \right|}$ is the associated Legendre polynomial of degree τ and order β.

In practice, of course, the SH series is truncated by discarding harmonics above degree *N*, yielding an *N*th order approximation. N=70 suffices to accurately model the surface of renal tumors. Finally, the renal tumor object is reconstructed from the SHs of Equation (3). The first few harmonics describe the rough extent of the tumor, while higher degree harmonics provide the finer details of its surface. Therefore, benign tumors are accurately represented by a lower-order SH model, while malignant tumors, with their more complex morphology, require higher-order SH model to describe their shape.

**Figure 4:**
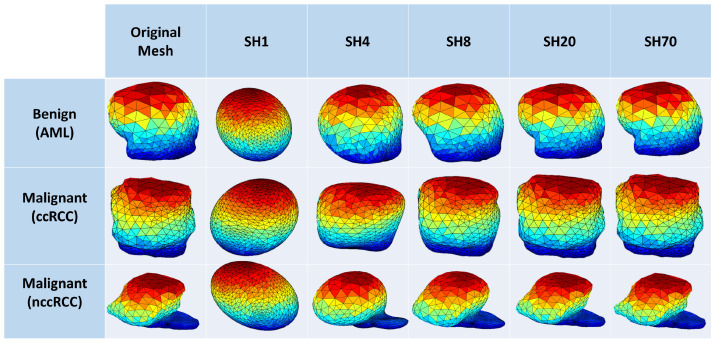
Renal tumors’ reconstruction meshes showing the morphological differences among malignant ccRCC, malignant nccRCC, and benign AML tumors.

Figure 4 shows the morphology approximation for three different renal tumors: malignant ccRCC, malignant nccRCC, and benign AML tumors. A summary of the attraction–repulsion algorithm is provided below.

**Initialization:**

- Triangulate the surface of the tumor.
- Smooth the triangulated mesh with Laplacian filtering.
- Initialize the spherical parameterization with an arbitrary, topology-preserving map onto the unit sphere.
- Fix values of $C_{A,1}$, $C_{A,2}$, $C_{R}$, and threshold *T*.

**Attraction–repulsion:**

- **For**  α=0,1,…
  ‒ **For**  i=1,…,I
    * Calculate $C_{\alpha +1,i}^{\prime }$ using Equation (1)
  ‒ **For**  i=1,…,I
    * Calculate $C_{\alpha +1,i}^{\prime \prime }$ using Equation (2)
    * Let $C_{\alpha +1,i}=C_{\alpha +1,i}^{\prime \prime }/∥C_{\alpha +1,i}^{\prime \prime }∥$
  ‒ **If**$\;\max_{i}∥C_{\alpha +1,i}-C_{\alpha ,i}∥\leq T$**Then**, let $\alpha_{f}=\alpha +1$, and **Stop**.

**Figure 5:**
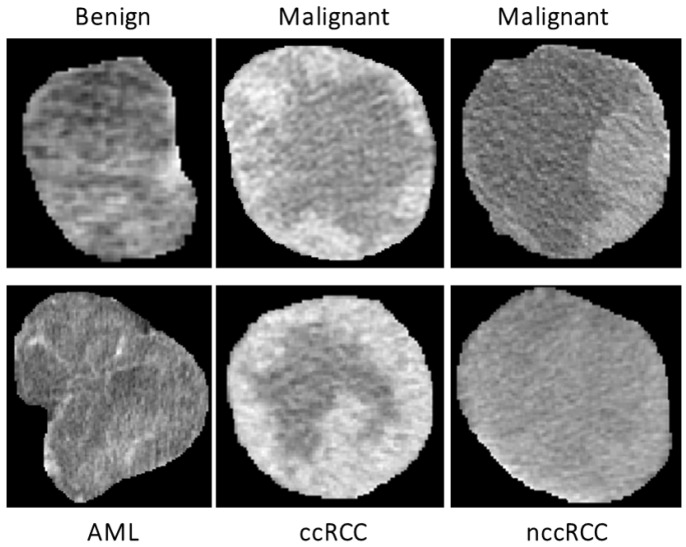
An illustrative example showing differences in texture between various renal tumor types.

**Textural features:** Recently, TA has become a popular research topic, particularly in the field of medical imaging. New techniques of TA provide different quantitative patterns/descriptors by combining the grey values of each pixel/voxel in a tumor image/volume. As a result of these abilities, TA has been used in the diagnosis of several tumors and their related subtypes with encouraging classification abilities [24,25,42,43,44,45,46,47,48]. Therefore, in this manuscript, TA techniques were applied on the segmented 3D renal tumor volumes to precisely extract first- and second-order textural features that best describe the homogeneity/heterogeneity between renal tumors with different diagnoses. The use of such comprehensive textural features relies on the fact that malignant tumors mostly show high textural heterogeneity when compared to benign ones. The success of these findings would enhance the sensitivity and the specificity towards an early identification of renal cancer tumors. Figure 5 demonstrates the lesion texture differences of two malignant ccRCC subjects, two malignant nccRCC subjects, and two benign (AML) subjects.

**First-order textural features:** These textural features include any quantity that can be derived from the gray-level histogram of the tumor volume. In particular, mean, variance, standard deviation, entropy, skewness, kurtosis, cumulative distribution functions, and the grey-level percentiles [49] were extracted.

**Figure 6:**
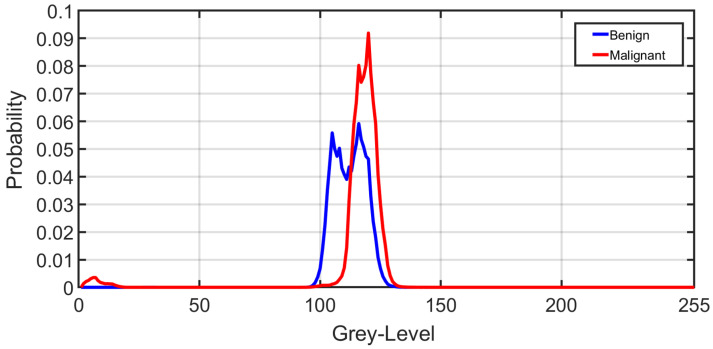
A visualization of the average normalized histogram curves for all benign subjects (blue) vs. malignant (red).

Figure 6 shows the average normalized histogram curves for all benign subjects (blue) vs. malignant (red). To construct these curves, the grey-level range was normalized first by dividing by the maximum grey-level value obtained from all subjects. Then, all histograms were constructed for all subjects within the new normalized grey-level range from 0 to 255. For each subject, the individual grey-level probability was obtained by dividing the histogram values by the corresponding number of voxels. Then, all normalized histograms from a particular group (malignant or benign) were averaged pointwise to obtain the final curve.

**Second-order textural features:** Since the first-order textural features might not be sufficient, with their range of values exhibiting significant overlap across classes, especially between subtypes of malignant tumor, second-order textural features were incorporated into the system. These features describe the joint distribution of gray values in multiple voxels that are considered to be neighbors of each other. In particular, the grey-level co-occurrence matrix (GLCM) [50] was used to capture the heterogeneous appearance of renal tumors.

**Figure 7:**
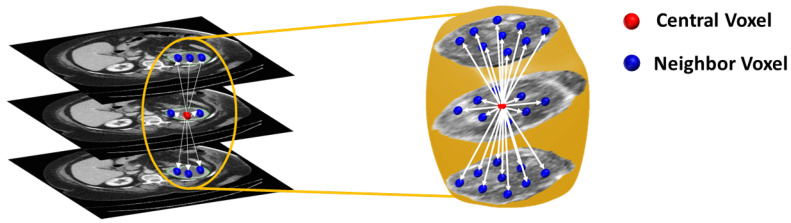
Visualization of the rotation-invariant neighborhood calculation system used to construct the grey-level co-occurrence matrix (GLCM). The GLCM can be constructed by counting the occurrence frequency of different grey-level pairs in-plane and in adjacent planes accounting for the 26-neighbor voxels (blue) of the central voxel (red).

To construct the GLCM, we must count the number of times an ordered pair of two grey values occurs in two neighboring voxels within the renal tumor object. This technique is continued until all conceivable occurrence frequencies within the grey-level range of the renal tumor item are found, which covers all possible pairs of neighbors. For this, we first contrast stretched the renal tumor object’s original grey-level range to fit the desired span 0–255, yielding a GLCM matrix with a size of 256 × 256. Then, all feasible pair combinations were identified to construct the GLCM matrix (i.e., neighbors with gray levels *i* and *j* contribute to row *i*, column *j* of the GLCM). To define our neighborhoods, we used a distance criterion that voxels must be separated by ≤$\sqrt{2}$ mm, making the calculations rotation invariant (see Figure 7). The resultant GLCM was then normalized and used to extracting the following second-order texture features [49,50]: contrast, dissimilarity, homogeneity, angular second moment (ASM), energy, and correlation.

The definitions of all first- and second-order textural features are provided in Table 1 and Table A1 in Appendix A.

**Figure 8:**
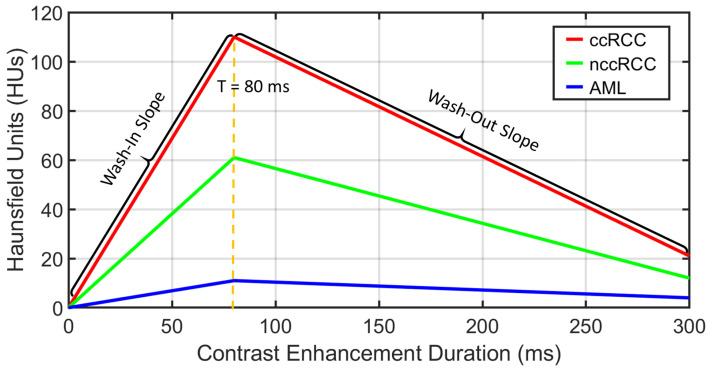
Example of the wash-in and wash-out slopes construction process for various types of renal tumors. When compared to nccRCC (green) and AML (blue), ccRCC tumors exhibit higher and faster wash-in/-out slopes (red).

**Functional features:** Discriminating RCC from AML, as well as ccRCC from nccRCC might be achieved using time-dependent characteristics of CE-CT imaging. The most relevant CE-CT findings for this purpose are generally homogenous and prolonged enhancement patterns [51]. The time dependency can be expressed by the slopes of wash-in and wash-out. Wash-in is described as the rate of increasing attenuation (in HU) from the precontrast to portal-venous phase. Similarly, wash-out is the rate of decrease in attenuation between the portal-venous and delayed-contrast phase [52]. Higher slopes of wash-in and wash-out are typically associated with malignancy. Moreover, nccRCC demonstrates wash-in and wash-out slopes intermediate between those of AML and those of ccRCC [53]. Therefore, we constructed both wash-in and wash-out slopes for all renal tumor subjects for the classification of the renal tumor status. Examples of wash-in/-out slopes showing the differences across ccRCC, nccRCC, and AML are shown in Figure 8.

### 3.3. Feature Integration and Renal Tumor Classification

Following the extraction of morphological, textural, and functional features from all given renal tumors, RC-CAD proceeds with two-stage diagnostic classification. The first stage aims to differentiate malignant (RCC) from benign (AML) tumors. In the case of malignancy, the second stage provides the classification of RCC tumors as ccRCC or nccRCC.

The multilayer perceptron (MLP) artificial neural network (ANN) consists of at least three layers: an input layer, one or more hidden layers, and an output layer, each with arbitrarily many activation/processing units, known as nodes/neurons. Each layer is fully connected to the next layer in sequence. Neurons use nonlinear activation functions to give the MLP-ANN the capability to divide the feature space into arbitrarily complex regions. The MLP-ANN mainly utilizes supervised backpropagation learning technique in the training phase, in which gradient descent methods are utilized to update the connection weights and additive biases in order to minimize the loss function. To achieve our goal, we utilized the MLP-ANN in both classification stages to obtain the final diagnosis. Classifier performance was assessed using five different feature sets (Table 2) as the ANN input in both stages. Feature Set 1 includes first-order histogram textural features (*N* = 6; mean, variance, standard deviation, skewness, kurtosis, and entropy); Feature Set 2 includes first-order percentile textural features (*N* = 10; from the 10th to the 100th percentile in 10% point steps); Feature Set 3 includes second-order GLCM textural features (*N* = 6; contrast, dissimilarity, homogeneity, ASM, energy, and correlation); Feature Set 4 includes SH reconstruction error (SHRE) morphological features (*N* = 70); and Feature Set 5 includes functional features (*N* = 2; wash-in slope and wash-out slope). At each classification stage, the individual feature sets were concatenated to obtain the combined features (*N* = 94) and were fed to a MLP-ANN to obtain the final diagnosis.

## 4. Results

The diagnostic performance of the RC-CAD system on our dataset of 140 renal tumors was assessed using leave-one-subject-out (LOSO) cross-validation. The system’s diagnostic capabilities were assessed, evaluated, and compared in both classification stages using the individual feature sets, as well as the combined features. Each classification process was repeated 10 times, and the results were tabulated in terms of the mean ± the standard deviation to provide a more quantitative expression of the diagnostic performance.

The first stage classification (RCC vs. AML) performance for the RC-CAD system was first evaluated using individual Feature Sets 1, 2, 3, 4, and 5 (see Table 2) along with different MLP-ANN classification models. Then, the RC-CAD system was evaluated using the combined features, resulting in a noticeably enhanced diagnostic performance. A summary of the first stage performance in terms of the sensitivity, specificity, and Dice similarity coefficient (DSC) [54,55] is presented in Table 3.

The diagnostic performance of the second stage classification (ccRCC vs. nccRCC) of the RC-CAD system was evaluated using the same LOSO cross-validation approach. As before, specially tailored MLP-ANN models were used with different feature sets. The best second stage classifier performance was obtained using the concatenated feature set (Table 4).

**Figure 9:**
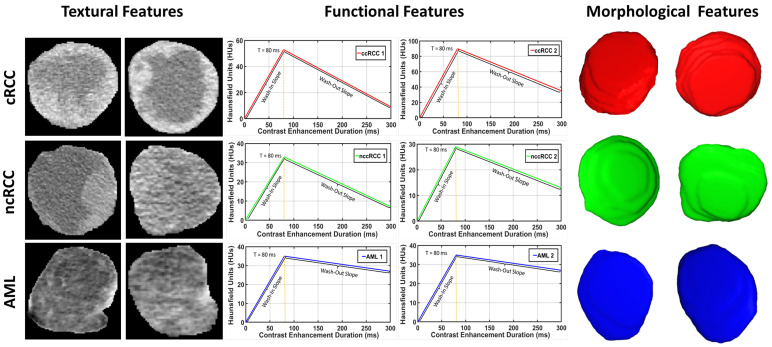
A difficult case presentation showing the textural differences, wash-in and wash-out slope differences, and shape differences between two ccRCC, two nccRCC, and two AML renal tumors.

Figure 9 demonstrates a difficult case presentation for two ccRCC, two nccRCC, and two AML renal tumors. This figure visualizes the texture differences, wash-in and wash-out slope differences, and morphological differences between the different types of renal tumors, which emphasizes the potential power of the integration process of such features in providing a precise identification of a given renal tumor.

To ensure that our system is not prone to overfitting and to validate the reproducibility and robustness of RC-CAD, we performed a randomly stratified 10-fold cross-validation approach in both stages using the combined features. Likewise, the classification process was repeated 10 times using the same MLP-ANN classification model, and the results are tabulated in terms of the mean ± the standard deviation (Table 5).

To highlight the advantages of using the MLP-ANN classifier, we compared RC-CAD with other, well-known machine learning classifiers (e.g., ${\mathrm{SVM}}_{Quad}$ and RF). As documented in Table 5, the diagnostic performance obtained by the developed RC-CAD system outperformed all other machine learning classifiers in both classification stages, which justifies the potential of such MLP-ANN classifiers being utilized for the developed RC-CAD system. It is worth mentioning that, in each classification stage, a grid search algorithm was employed to find the optimal set of hyperparameters, with the classification accuracy optimization criterion, for each of the classifier techniques being evaluated. The results of the hyperparameter optimization are appended to Table 5.

For the comparison with RC-CAD, we applied the existing state-of-the-art approach [27] using a total of 10 textural markers extracted from the portal-venous phase only along with the gradient boosting classification technique. In addition, we applied the state-of-the-art deep learning CNN approaches proposed by Lee et al. [32] and Oberai et al. [37] on our own datasets (first stage: N = 140; second stage: N = 70). To highlight the advantages of the RC-CAD system, all results are compared in Table 6. The diagnostic performance of RC-CAD exceeded that of other approaches in both classification stages.

## 5. Discussion

The developed RC-CAD system demonstrated high diagnostic performance in terms of accuracy, sensitivity, specificity, and DSC in discrimination between benign (AML) and malignant (RCC) and in classification of the RCC subtype into ccRCC or nccRCC. This early and precise identification of the malignancy status of a given renal tumor and its associated subtype can enable clinicians to provide the appropriate early intervention/treatment plan and improve the outcomes. CE-CT was utilized as it is an imaging modality with the ability to provide different aspects of features, including but not limited to, morphological features, textural features, and functional features. The integration of these features is effective in determining the malignancy status of a given renal tumor when combined with a powerful machine learning classifier such as the MLP-ANN.

The grade of malignancy of a given renal tumor largely specifies the morphology of the tumor. Typically, malignant tumors demonstrate a more complex morphology than benign ones. Therefore, morphological features based on using spherical harmonics were utilized to capture possible surface complexity differences between malignant and benign renal tumors, as well as differences between different subtypes of malignancy.

First- and second-order textural features have been widely utilized to identify a given renal tumor status as malignant or benign, as well as to describe the malignancy subtype [26,27,29,31,32,38]. These features capture all possible textural homogeneity/heterogeneity across renal tumors with different diagnoses. In line with these studies, the extracted textural features provided high diagnostic performance in discriminating malignant ccRCC and nccRCC from benign (AML) renal tumors.

Additionally, functionality was utilized in identifying the malignancy status of a given renal tumor. The slopes of wash-in and wash-out can capture the existing differences in the enhancement characteristics [51,52]. In this study, the results obtained by the functionality metrics demonstrated the efficacy of such features in discriminating between benign (AML) and malignant (RCC) and identifying the malignancy subtype as ccRCC or nccRCC.

Although individual features have provided a reasonable diagnostic performance, they are not sufficient to rule out surgical intervention in (what may turn out to be) benign lesions. Therefore, the integration process of these features is critical to enhance the diagnostic accuracy to the point of clinical utility. The integration process produced a reliable and accurate RC-CAD system with an enhanced diagnostic performance in both classification stages as documented in Table 3, Table 4 and Table 5.

This study has some limitations: (i) benign tumors only included AMLs and did not include any ONCs; (ii) the datasets in this study were all collected from the same geographical area, and thus, we did not account for population diversity; (iii) demographics such as age and sex were not included in our analysis; (iv) differentiation between paRCC and chrRCC was not performed due to the limited number of subjects; and (v) the RC-CAD system in its current form still requires expert knowledge to segment the renal tumor manually before the handcrafted features are extracted. Despite these limitations, the RC-CAD system demonstrated the efficacy and feasibility of integrating various types of features to account for different aspects, making the developed RC-CAD a reliable noninvasive diagnostic tool.

## 6. Conclusions and Future Work

The developed RC-CAD system demonstrated a high classification sensitivity of 95.29%±2.03%, a specificity of 99.86%±0.43%, an ad DSC of 0.98±0.01 in differentiating benign AML from malignant RCC renal tumors. In addition, the RC-CAD achieved an overall classification accuracy of 89.57%±5.03% in distinguishing ccRCC from nccRCC to provide the proper management plan. Integrating accurate morphological features with functional features and multiple first-order and second-order textural features was adequate to significantly enhance the diagnostic capabilities. Future work will obtain data from a larger cohort spanning different geographical areas to test the RC-CAD system’s generalizability. In addition, new types of renal tumors including oncocytomas and malignant lymphomas will be included to expand the subclassification abilities of the RC-CAD system. This greater amount of data will necessitate a fully automated segmentation approach to be incorporated into the system, as manual segmentation will become too burdensome. Furthermore, fully automated extraction of diagnostic image features might be achieved using state-of-the-art deep learning approaches (e.g., convolutional neural networks and stacked auto-encoders).

## Figures and Tables

**Table 1:** Definition of first- and second-order textural features.

Textural Feature	Definition
**First-Order**	
Mean	The average grey value of voxels within the tumor.
Variance	Second central moment of gray values.
Standard deviation	Square root of variance.
Skewness (Skew)	Asymmetry of the distribution of gray values about the mean. If Skew < 0, that means the grey level spreads out more to the left of the mean than to the right, and if Skew > 0, that means the grey level spreads out more to the right of the mean than to the left. Skew will equal zero in the case of normal distributions.
Kurtosis (Kurt)	Measures the tail weight, or tendency to extreme values, of the object grey-level distribution. The normal distribution has Kurt = 3; distributions with heavier tails have Kurt > 3; distributions with less weight in the tails have Kurt < 3.
Entropy	A measure of randomness of grey values within an input image.
CDFs	A distribution function that accumulates voxel-wise grey values from the whole tumor object with minimum value = 0 and maximum value = 1.
Percentiles	Grey values percentiles corresponding to the CDFs (from 10% to 100%)
**Second-Order**	
Contrast	Measures the disparity in grey-level values between neighbors.
Dissimilarity	Finds to what extent voxels are different from their neighbors.
Homogeneity	Expresses the inverse difference moment among neighbors.
Angular second moment (ASM)	Determines the gray levels’ local uniformity (orderliness).
Energy	The square root of the ASM.
Correlation	Determines the grey-level linear dependency in neighborhood blocks.

**Table 2:** Details of the extracted feature sets used in the two-stage renal tumor classification.

**Texture Features**	
Feature Set 1: First-order (histogram features)	6 features
Feature Set 2: First-order (percentiles)	10 features
Feature Set 3: Second-order (GLCM)	6 features
**Shape Features**	
Feature Set 4: Spherical harmonic reconstruction errors	70 features
**Functional Features**	
Feature Set 5: Wash-in/out slopes	2 features
**Combined Features**	
Feature Sets 1, 2, 3, 4, and 5	94 features

**Table 3:** Diagnostic performance results of the first stage classification (RCC vs. AML) using different individual feature sets along with multilayer perceptron artificial neural network (MLP-ANN) classification models. The RC-CAD system diagnostic performance using the combined features outperformed the diagnostic abilities using individual feature sets. Sens: sensitivity, Spec: specificity, DSC: Dice coefficient of similarity, ${\mathrm{hl}}_{n}$: size of hidden layer *n*.

RCC vs. AML Classification Performance (Mean ± SD ≈)				
**Feature Set**	**Sens%**	**Spec%**	**DSC**	**MLP-ANN**
Set 1	94.1 ± 1.5	97.9 ± 1.5	0.96 ± 0.01	hl${}_{1}$ = 10 nodes
Set 2	92.4 ± 2.9	95.1 ± 3.5	0.94 ± 0.02	hl${}_{1}$ = 10 nodes
Set 3	94.9 ± 2.2	95.3 ± 2.5	0.95 ± 0.02	hl${}_{1}$ = 10 nodes
Set 4	92.0 ± 2.4	96.6 ± 2.0	0.94 ± 0.02	hl${}_{1}$ = 10 nodes, hl${}_{2}$ = 5 nodes
Set 5	82.7 ± 4.1	91.7 ± 2.0	0.87 ± 0.02	hl${}_{1}$ = 10 nodes
**RC-CAD**	**95.3 ± 2.0**	**99.9 ± 0.4**	**0.98 ± 0.01**	**hl${}_{1}$ = 50 nodes, hl${}_{2}$ = 25 nodes**

Hyperparameters: MLP-ANN (optimization function: trainlm, max epochs = 500, goal = 0, max validation failure = 6, min gradient = $10^{-7}$, training gain (μ): initial μ = 0.001, μ decrease factor = 0.1, μ increase factor = 10, max μ = 1e${}^{10}$).

**Table 4:** Results from the second stage classification (ccRCC vs. nccRCC) using individual feature sets (1, 2, 3, 4, and 5) along with the multilayer perceptron artificial neural network (MLP-ANN) classification models. The RC-CAD system diagnostic performance using the combined features outperformed the diagnostic abilities using individual feature sets. Acc: accuracy, hl${}_{n}$: size of hidden layer *n*.

ccRCC vs. nccRCC Classification Performance (Mean ± SD ≈)		
**Feature Set**	**Acc%**	**MLP-ANN Architecture**
Set 1	76.8 ± 2.6	hl${}_{1}$ = 10 nodes
Set 2	75.7 ± 3.8	hl${}_{1}$ = 10 nodes
Set 3	83.3 ± 5.6	hl${}_{1}$ = 10 nodes
Set 4	81.4 ± 5.1	hl${}_{1}$ = 10 nodes, hl${}_{2}$ = 5 nodes
Set 5	76.2 ± 2.33	hl${}_{1}$ = 10 nodes
**RC-CAD**	**89.6 ± 5.0**	**hl${}_{1}$ = 50 nodes, hl${}_{2}$ = 25 nodes**

Hyperparameters: MLP-ANN (optimization function: trainlm, max epochs = 500, goal = 0, max validation failure = 6, min gradient = $10^{-7}$, training gain (μ): initial μ = 0.001, μ decrease factor = 0.1, μ increase factor = 10, max μ = 1e${}^{10}$).

**Table 5:** Diagnostic performance comparison for both classification stages between the developed RC-CAD system and other classification approaches (e.g., random forest (RF) and support vector machine (SVM)). Using leave-one-subject-out (LOSO) and a randomly stratified 10-fold cross-validation approach, the diagnostic abilities of the RC-CAD outperformed the others. Let Sens: sensitivity, Spec: Specificity, DSC: Dice similarity coefficient, and Acc: Accuracy.

**First Stage Classification (RCC vs. AML) Performance (Mean ± SD ≈)**				
**Method**	**Validation**	**Sens%**	**Spec%**	**DSC**
**RC-CAD (Proposed)**	**LOSO**	**95.3 ± 2.0**	**99.9 ± 0.4**	**0.98 ± 0.01**
	**10-fold**	**89.0 ± 3.4**	**91.0 ± 2.7**	**0.90 ± 0.02**
RFs	LOSO	89.0 ± 1.7	92.7 ± 2.7	0.91 ± 0.02
	10-fold	88.4 ± 1.0	90.7 ± 3.0	0.89 ± 0.01
SVM${}_{Quad}$	LOSO	82.9 ± 0.0	88.6 ± 0.0	0.85 ± 0.00
	10-fold	81.9 ± 2.2	87.7 ± 2.5	0.84 ± 0.02
**Second Stage Classification (ccRCC vs. nccRCC) Performance (Mean ± SD ≈)**				
**Method**	**Validation**		**Acc%**	
**RC-CAD (Proposed)**	**LOSO**		**89.6 ± 5.0**	
	**10-fold**		**78.6 ± 5.7**	
RFs	LOSO		53.7 ± 3.7	
	10-fold		51.9 ± 2.6	
SVM${}_{Quad}$	LOSO		52.9 ± 0.0	
	10-fold		54.3 ± 3.0	

Hyperparameters: MLP-ANN (optimization function: trainlm, max epochs = 500, hidden layers: hl${}_{1}$ = 50 nodes, hl${}_{2}$ = 25 nodes, goal = 0, max validation failure = 6, min gradient = $10^{-7}$, training gain (μ): initial μ = 0.001, μ decrease factor = 0.1, μ increase factor = 10, max μ = 1e${}^{10}$); RF (method: Bag, number of learning cycles = 30); SVM (kernel function: quadratic, box constraint = 1).

**Table 6:** Diagnostic performance comparison for both classification stages between the developed RC-CAD system and the state-of-the-art approaches by [27,32,37]. The diagnostic abilities of the RC-CAD outperformed all other methods in both classification stages. Let Sens: sensitivity, Spec: Specificity, DSC: Dice similarity coefficient, and Acc: Accuracy.

**First Stage Classification (RCC vs. AML) Performance (Mean ± SD ≈)**				
**Method**		**Sens%**	**Spec%**	**DSC**
**RC-CAD (Proposed)**		**95.3 ± 2.0**	**99.9 ± 0.4**	**0.98 ± 0.01**
Kunapuli [27]		81.4 ± 0.0	95.7 ± 0.0	0.88 ± 0.00
Oberai [37]		88.9 ± 1.7	87.4 ± 1.4	0.91 ± 0.01
Lee [32]	AlexNet	84.0 ± 1.7	93.4 ± 1.9	0.88 ± 0.02
	GoogleNet	88.3 ± 1.7	95.1 ± 1.9	0.91 ± 0.01
	ResNet	88.0 ± 3.5	95.7 ± 0.9	0.91 ± 0.02
	VGGNet	86.9 ± 0.6	91.4 ± 2.4	0.89 ± 0.01
**Second Stage Classification (ccRCC vs. nccRCC) Performance (Mean ± SD ≈)**				
**Method**		**Acc%**	**ccRCC/40**	**nccRCC/30**
**RC-CAD (Proposed)**		**89.6 ± 5.0**	**35 ± 2**	**28 ± 3**
Kunapuli [27]		60.6 ± 2.7	28 ± 1	15 ± 1
Oberai [37]		84.3 ± 3.1	34 ± 1	25 ± 2
Lee [32]	AlexNet	71.7 ± 1.9	31 ± 2	19 ± 2
	GoogleNet	68.0 ± 1.5	32 ± 1	15 ± 1
	ResNet	70.3 ± 2.5	32 ± 0	17 ± 2
	VGGNet	72.6 ± 2.3	33 ± 1	18 ± 1

Hyperparameters: MLP-ANN (optimization function: trainlm, max epochs = 500, hidden layers: hl${}_{1}$ = 50 nodes, hl${}_{2}$ = 25 nodes, goal = 0, max validation failure = 6, min gradient = $10^{-7}$, training gain (μ): initial μ = 0.001, μ decrease factor = 0.1, μ increase factor = 10, max μ = 1e${}^{10}$).

## Data Availability

Data could be made available after acceptance upon a reasonable request to the corresponding author.

## Appendix A

In this Appendix, we detail the mathematical formulas used to extract the textural features:

Basic notation:

- μ: mean;
- *n*: total number of voxels in the object;
- $v_{i}$: gray-level value of Voxel *i*;
- $\sigma^{2}$: variance;
- σ: standard deviation;
- $N_{g}$: the normalized grey levels;
- *p*: the normalized histogram counts;
- ϵ: an initial random small number;
- $N_{g}$: grey-levels (normalized 0–255);
- $G_{N}$: the GLCM (normalized 0–1);
- $\bar{x}$, $\sigma_{x}\left(i\right)$: the row margins (mean and standard deviation);
- $\bar{y}$, $\sigma_{y}\left(i\right)$: the column margins (mean and standard deviation).

**Table A1:** Texture features formulas.

Feature	Formula
**First-Order**	
Mean (μ)	(A1) $\frac{1}{n}\sum_{i=1}^{n}v_{i}=\frac{v_{1}+v_{2}+\cdots +v_{n}}{n}$
Variance ($\sigma^{2}$)	(A2) $$\frac{\sum_{i=1}^{n}{\left(v_{i}-\mu \right)}^{2}}{n}$$
Entropy (Ent)	(A3) $-\sum_{i=1}^{N_{g}}p\left(i\right)\log_{2}\left(p(i)+\epsilon \right)$
Skewness (Skew)	(A4) $\frac{1}{n}\sum_{i=1}^{n}{\left(\frac{v_{i}-\mu }{\sigma }\right)}^{3}$
Kurtosis (Kurt)	(A5) $\frac{1}{n}\sum_{i=1}^{n}{\left(\frac{v_{i}-\mu }{\sigma }\right)}^{4}$
**Second-Order**	
Contrast	(A6) $\sum_{i=0}^{N_{g}}\sum_{j=0}^{N_{g}}{\left(i-j\right)}^{2}G_{N}\left(i,j\right)$
Dissimilarity	(A7) $\sum_{i=0}^{N_{g}}\sum_{j=0}^{N_{g}}\left|i-j\right|G_{N}\left(i,j\right)$
Homogeneity	(A8) $\sum_{i=0}^{N_{g}}\sum_{j=0}^{N_{g}}\frac{G_{N}\left(i,j\right)}{1+{\left(i-j\right)}^{2}}$
ASM	(A9) $\sum_{i=0}^{N_{g}}\sum_{j=0}^{N_{g}}{\left(G_{N}\left(i,j\right)\right)}^{2}$
Energy	(A10) $\sqrt{ASM}$
Correlation	(A11) $\frac{\sum_{i=0}^{N_{g}}\sum_{j=0}^{N_{g}}G_{N}\left(i,j\right)ij-\bar{x}\bar{y}}{\sigma_{x}\left(i\right)\sigma_{y}\left(j\right)}$
